# Electrophysiological and morphological alteration in the visual pathway of children with attention-deficit/hyperactivity disorder

**DOI:** 10.3389/fpsyt.2026.1803834

**Published:** 2026-04-27

**Authors:** Zixuan Huang, Ye Wu, Gantian Huang, Qian Chen, Dapeng Chen, Hui Zhou, Longqian Liu

**Affiliations:** 1 Department of Ophthalmology, West China Hospital, Sichuan University, Chengdu, China; 2 Laboratory of Optometry and Vision Sciences, West China Hospital, Sichuan University, Chengdu, Sichuan, China; 3 Department of Optometry and Vision Sciences, West China School of Medicine, Sichuan University, Chengdu, Sichuan, China; 4 Center of Biostatistics, Design, Measurement and Evaluation, Department of Clinical Research Management, West China Hospital of Sichuan University, Chengdu, Sichuan, China; 5 Department of Rehabilitation Medicine, Key Laboratory of Birth Defects and Related Diseases of Women and Children, Ministry of Education (MOE), West China Second University Hospital, Sichuan University, Chengdu, Sichuan, China

**Keywords:** attention-deficit/hyperactivity disorder, electrophysiology, optical coherence tomography, visual evoked potentials, visual pathway

## Abstract

**Introduction:**

Attention-deficit/hyperactivity disorder (ADHD) is one of the most common neurodevelopmental disorders in children. Optical Coherence Tomography (OCT) and Visual evoked potentials (VEP) are common non-invasive diagnostic techniques. Researchers can use these techniques to identify possible biomarkers and explore the neurodevelopmental mechanisms underlying ADHD.

**Methods:**

The ADHD group (37 cases, average age 8.81 ± 1.44 years) and the healthy controls (38 cases, average age 8.97 ± 1.43 years), had the OCT and VEP. The retinal nerve fibre layer (RNFL), optic disc parameters, and macular parameters were measured through OCT. The latencies of P100 and the amplitudes of N75-P100 and P100-N135 waves at three different spatial frequencies (visual angles of 15’, 30’, and 60’) were tested through VEP.

**Results:**

The average RNFL and RNFL in each quadrant between the two groups were no statistically significant (all *p* > 0.05). The optic disc area, average cup-to-disc ratio, and cup volume in the ADHD group were all significantly larger than those in the control group (all *p* < 0.05). At three visual angles (15’, 30’, 60’), P100-latency in the ADHD group were all more significant than those in the control group (all *p* < 0.05). The amplitudes of N75-P100 and P100-N135 in the ADHD group were all statistically significantly lower than those in the control group (all *p* ≤ 0.001).

**Discussion:**

From the perspective of electroencephalophysiology, children with ADHD may have early visual information processing disorders. This provides a theoretical and practical basis for further early intervention in children with ADHD from the field of visual perception. The study protocol followed the tenets of the Declaration of Helsinki, was approved by the local ethics committee (No 2023-2240), and was registered on ClinicalTrials.gov (ChiCTR2400086223).

## 1 Introduction

Attention-deficit/hyperactivity disorder (ADHD) is a common neurodevelopmental disorder, with an estimated prevalence between 6.7% and 7.8% (1). Children patients mainly present with difficulties in maintaining friendships with peers, challenges in completing their studies, and a decline in self-esteem (2). Although ADHD is becoming increasingly common in children, its diagnosis still relies on clinical assessment (3). Previous studies have shown that objective biological markers are very important for the research of such neurodevelopmental disorders. Since both the retina and the cerebral cortex originate from the neural ectodermal cells of the neural crest, the retina is similar to the brain and spinal cord in many aspects (4, 5). Therefore, it is possible to explore objective biomarkers related to ADHD from the retina and the optic nerve.

Optical Coherence Tomography (OCT) is a non-invasive diagnostic technique. It has been widely used in the research of various ophthalmic diseases (6, 7). Moreover, OCT has been applied in the research of some neuropsychiatric diseases, such as Alzheimer’s disease (8, 9), multiple sclerosis (10), Parkinson’s disease (11), Specific Learning Disorder (12), Schizophrenia (13, 14), autism spectrum disorders (15), and bipolar disorder (16). For example, the OCT results of Alzheimer’s disease patients noted a significant structural change (8). Although the changes in ocular manifestations are not specific to a particular disease, this indicates a close connection between the retina and the brain. In recent years, scholars have proposed that the retinal characteristics of patients with neurodevelopmental disorders also differ from those of normal participants (17). Although a large number of studies have examined the brain structure and function of patients with ADHD through brain imaging (18), however, it is not suitable for routine clinical screening of children with ADHD due to the high time cost, high economic cost and inconvenient operation of magnetic resonance scanners. Considering the existing connection between the brain and the retina, OCT may become a particularly attractive and simple research method for children with ADHD (4, 19–21). Li et al. (22) found that the overall thickness of the retinal nerve fibre layer (RNFL) in ADHD groups decreased slightly compared with healthy controls. However, Sima et al. (12) found that there were no significant differences in the retinal parameters between ADHD children and healthy controls. Due to the differences in the races of the participants, the examination methods, the severity of the disease, and the sample sizes in current studies, all these factors may lead to inconsistent results among different studies. Overall, the application of OCT may contribute to understanding the neurobiological mechanisms underlying ADHD. More research is needed for further exploration.

Visual evoked potentials (VEP) is a non-invasive recording of cortical neural activity that does not require participants to make verbal or behavioural responses, so it can be used for children with neurodevelopmental disorders. The amplitude of VEP is correlated with the impairment of higher cognitive functions in children with neurodevelopmental disorders (23–25). Numerous studies have shown that there are abnormalities in the latency or amplitude of the VEP P100 wave in certain mental and neurological disorders, such as in schizophrenia (26, 27), multiple sclerosis (28, 29), and epilepsy (30). Some studies even suggest that the characteristics of VEP signals can serve as effective discriminative indicators for mental disorders (31). Currently, only a few studies have attempted to conduct research on patients with ADHD through VEP. Adeleh et al. (32) found that children with ADHD could elicit a higher P100 amplitude than children with Bipolar Mood Disorder, and the amplitudes of these two groups were different from those of healthy controls. However, this study found that there was no statistically significant difference in the P100 latency between children with ADHD and healthy controls. Alaa et al. (32) found that both children with Autism Spectrum Disorder (ASD) and those with ADHD showed more significant delays in the latency of the P100 wave than healthy controls. In summary, research on analysing the characteristics of VEP signals in patients with ADHD is still limited, and different participants and various visual stimuli were used in each study. When the spatial frequency of visual stimuli is higher, the visual information becomes more complex. If children with ADHD have more difficulty in processing more complex visual information, it maybe manifested as a slower conduction velocity of nerve fibres and a more significant prolongation of the P100 wave latency. These are questions that existing academic research cannot answer at present and await further research and exploration.

Previous studies have shown that the combined application of VEP and OCT can provide important evidence for the assessment of the visual pathway. Eklund et al. (33) comprehensively evaluated the visual pathway of patients with secondary progressive multiple sclerosis using a combination of VEP and OCT, and found that the thickness of the inner plexiform layer of retinal ganglion cells and the RNFL were significantly negatively correlated with the latency of the VEP P100 wave. Daina et al. (34) confirmed that there was a significant positive correlation between the RNFL thickness and the amplitude of the VEP P100 wave in multiple sclerosis patients. OCT can indirectly evaluate the functional integrity of optic nerve axons and the situation of neuronal loss, while VEP can effectively reflect the functional damage of the post-retinal visual pathway nerve structure (35). Existing evidence shows that these two non-invasive examination techniques can provide complementary information when evaluating the integrity of the visual pathway. In conclusion, this study intends to combine VEP and OCT techniques to systematically evaluate the structural and functional characteristics of the visual pathway in children with ADHD, in order to provide a new perspective for clarifying the overall pathological mechanism of ADHD from the perspective of the visual pathway.

Accordingly, this exploratory study was conducted to (1) measure the RNFL, optic disc parameters, and macular parameters of all the participants; (2) measure the latencies and amplitudes at three different spatial frequencies (visual angles of 15’, 30’, 60’) through VEP of all the participants; (3) compare the differences between ADHD group and control group based on the scores of the Swanson, Nolan, and Pelham Rating Scale–Fourth version (SNAP-IV). scale, to explore the neurodevelopmental mechanisms underlying ADHD.

## 2 Methods

### 2.1 Study participants

This study included two groups of participants in total: the ADHD group (37 cases, 74 eyes) and the control group (38 cases, 76 eyes). The binocular data of the participants were included in the statistical analysis. All children with ADHD were recruited from the Outpatient Clinic of Paediatric Rehabilitation Medicine, West China Second University Hospital, Sichuan University, between September 2023 and December 2024. The diagnosis was made by paediatric psychiatrists through a structured diagnostic interview based on the diagnostic criteria for ADHD in the Diagnostic and Statistical Manual of Mental Disorders, Fifth Edition (DSM-5) (36). The control group consisted of children who came to the Ophthalmology and Optometry Clinic of West China Hospital of Sichuan University during the same period for routine eye examinations. Their gender, age, and educational level were matched with those of ADHD group. All sociodemographic data were collected by physicians through interviews with parents.

Inclusion criteria of ADHD group comprised: (1) aged 7–12 years; (2) no intellectual disability, with a Full Scale Intelligence Quotient (FIQ) score of ≥ 80 on the Chinese Revision of the Wechsler Intelligence Scale for Children-IV; (3) having received formal school education under the same conditions and for the same duration; (4) right-handed. Exclusion criteria comprised: (1) history of previous brain injury; (2) other neurodevelopmental disorders and psychotic disorders; (3) receiving ADHD-related drug treatment or other non-drug intervention methods during the study period.

Inclusion criteria of healthy controls comprised: (1) aged 7–12 years; (2) never suffered from ADHD and have no past history of ADHD or other mental illnesses; (3) right-handed; (4) BCVA of 1.0 or above in both eyes, and normal accommodation and binocular vision functions. Exclusion criteria comprised: (1) history of previous brain injury;(2) other neurodevelopmental disorders and psychotic disorders; (3) strabismus or other organic eye diseases; (4) history of eye trauma or extraocular muscle surgery.

### 2.2 Study procedure

#### 2.2.1 OCT image acquisition

The OCT images were acquired using a Cirrus HD-OCT 5000 optical coherence tomography scanner (Carl Zeiss, Germany). Scan the thickness of the ganglion cell layer in the macular area, and simultaneously measure the retinal thickness and average thickness in the macular area and the four quadrants (superior, inferior, nasal, and temporal) around the optic disc. According to the grid division criteria of the Early Treatment Diabetic Retinopathy Study (37), parameters such as the foveal thickness and macular volume are obtained through computer processing technology. This study analysed the participants’ average RNFL thickness of the optic disc, the RNFL thickness in four quadrant areas (superior, inferior, nasal, and temporal), optic disc parameters (rim area, optic disc area, average cup-to-disc ratio, cup volume), and macular parameters (average macular thickness, central macular retinal thickness, and retinal thickness of the inner and outer rings). Assessors were blinded to diagnostic status during acquisition, The order of testing and whether standardized procedures were followed across participants.

#### 2.2.2 VEP signal acquisition

A visual electrophysiological examination system (RETI-port/scan 21, Roland Berger, Germany) was used to collect monocular full-field pattern-reversal VEP signals from all participants. The recording conditions comply with the VEP standards established by the International Society for Clinical Electrophysiology of Vision (ISCEV). The average luminance in the laboratory is 80 cd/m². The participants wear corrective glasses, keep their natural pupils. In this study, three checkerboards with three different visual angles (15’, 30’, and 60’) were used. In each recording, 100 scans are averaged. All participants were examined in the left and right eyes in a random order. The unexamined eye was covered, and an appropriate rest was allowed when changing the eye. Each eye was measured three times repeatedly. This study mainly analyses the latency of the P100 wave and the amplitudes of N75-P100 and P100-N135 waves in the participants. Assessors were blinded to diagnostic status during acquisition, The order of testing and whether standardized procedures were followed across participants.

#### 2.2.3 SNAP-IV scale

The SNAP-IV consists of 26 items that are rated on a 4-point scale (not at all, just a little, quite a bit, very much). The items are divided between three subscales: inattention (nine items), hyperactivity/impulsivity (nine items), and oppositional (eight items). Subscale scores are calculated by creating an average. Items for inattention and hyperactivity/impulsivity can be combined to also create a "combined ADHD" score. Higher scores represent more problem symptoms. The SNAP-IV was a measure used to assess ADHD symptoms in our research. The SNAP-IV was completed by parents online or on paper, and took approximately 15 minutes to complete.

### 2.3 Statistical analysis

In this study, IBM SPSS 27.0 statistical software was used for statistical analysis. Continuous variables (measurement data) conforming to the normal distribution were expressed as mean ± standard deviation. Continuous variables (measurement data) that do not conform to the normal distribution are presented as the median (range). Categorical variables were statistically described using frequency (percentage). We found that there was no statistical interocular difference between the two eyes of all participants (t = 1.505, P = 0.137), and previous studies have all included binocular data for the final comparative analysis (38–40), our research also included binocular data for the final comparative analysis. Before comparing between groups, a normality test is first performed. For data conforming to the normal distribution, the independent-samples t-test was used, while for data with non-normal distribution, the non-parametric Mann-Whitney U test was employed. Subsequently, a bivariate correlation analysis was performed between the P100 wave latency, N75-P100 wave amplitude, and P100-N135 wave amplitude of the ADHD group and the SNAP-IV rating scale scores. Pearson correlation analysis was used for data conforming to a normal distribution, and Spearman correlation analysis was used for non-normally distributed data. The Fisher r-to-z transformation analysis was used for the analysis of differences between correlation coefficients. In all statistical analyses of this study, *p* < 0.05 was considered statistically significant.

## 3 Results

### 3.1 Characteristics of the participants

This study comprised 37 ADHD children and 38 healthy controls, and their baseline demographic data are summarized in **Table 1**. There were no statistically significant differences between the two groups of participants in terms of gender, age, spherical equivalent, intraocular pressure, BCVA, etc. (all *p* > 0.05). The total score of the SNAP-IV scale in the ADHD group, as well as the scores in the two domains of inattention and hyperactivity/impulsivity, were all statistically significantly higher than those in the normal group (all *p* < 0.001) (**Table 1**).

**Table 1:** Comparison of characteristics between the ADHD group and the control group.

Variables	The ADHD group (n=37)	The control group (n=38)	t-value	p-value
Age (year)	8.81±1.44	8.97±1.43	0.694	0.489 a
Gender (boy/girl)	23/14	20/18	0.696	0.404 b
Spherical equivalent (D)	-1.36±1.77	-1.79±1.10	-1.786	0.076 a
IOP (mmHg)	15.54±2.43	14.98±2.20	-1.499	0.136 a
BCVA	0.003±0.02	0.00±0.00	-1.443	0.151 a
SNAP-IV scale scores				
Inattention	17 (3-27)	6.5 (0-22)	-8.372	<0.001 c
Hyperactivity/impulsivity	8 (0-25)	4 (0-16)	-5.072	<0.001 c
Oppositional defiant disorder	6 (0-21)	5 (1-11)	-1.299	0.194 c
Total score of the scale	31 (9-70)	17 (2-49)	-6.870	<0.001 c

SNAP-IV, swanson, nolan, and pelham-IV rating scales. Measurement data with normal distribution are expressed as "mean ± standard deviation"; measurement data with non-normal distribution are expressed as "median (range)"; count data are expressed as frequencies. a represents the independent samples t-test, b represents the chi-square test, and c represents the Mann-Whitney U test.

### 3.2 Comparison of peripapillary nerve fibre layer thickness and macular parameters between the two groups

An independent samples t-test was used to compare the average RNFL thickness, the RNFL thickness in the superior, inferior, temporal, and nasal quadrants of the optic disc, and other parameters between the ADHD group and the control group. The results showed no statistical significance (all *p* > 0.05). The rim area in the ADHD group was smaller than that in the control group, but the difference was not statistically significant (*p* = 0.360). However, the optic disc area, average cup-to-disc ratio, and cup volume in the ADHD group were all statistically significantly larger than those in the control group (all *p* < 0.05) (**Table 2**).

**Table 2:** Comparison of peripapillary nerve fibre layer thickness and optic disc parameters between the two groups and the control group.

Variables	The ADHD group	The control group	*t*-value	*p*-value	*Cohen’s d* (95% CI)
Average RNFL (μm)	102.27±10.99	102.11±8.55	-0.103	0.918 a	-0.017 (-0.337-0.303)
Superior quadrant of the Optic disc (μm)	133.32±17.68	134.26±17.17	0.330	0.742 a	0.054 (-0.266-0.374)
Inferior quadrant of the Optic disc (μm)	130.66±24.69	130.32±15.21	-0.104	0.918 a	-0.017 (-0.337-0.303)
Nasal quadrant of the Optic disc (μm)	69.89±14.78	66.21±10.02	-1.790	0.075 a	-0.292 (-0.614-0.030)
Temporal quadrant of the Optic disc (μm)	75.35±13.41	78.05±9.95	1.404	0.162 a	0.229 (-0.092-0.550)
Area of the disc rim	1.455 (0.98-2.44)	1.485 (1.22-2.06)	-0.916	0.360 b	
Optic disc area	2.000 (1.21-3.33)	1.860 (1.26-2.72)	-3.092	0.002 b	
Average cup-to-disc ratio	0.51 (0.07-0.71)	0.405 (0.07-0.70)	-3.177	0.001 b	
Optic cup volume	0.138 (0.00-0.61)	0.062 (0.00-0.46)	-2.658	0.008 b	
Retinal GCL+IPL thickness	84.15±5.26	83.68±4.04	-0.607	0.544 a	-0.099 (-0.419-0.221)
Average macular thickness (μm)	280.96±10.58	277.53±10.95	-1.953	0.053 a	-0.319 (-0.614-0.004)
Retinal thickness in the Central macular area (μm)	238.72±23.29	238.04±16.90	-0.204	0.839 a	-0.033 (-0.353-0.287)
Inner retinal thickness (μm)	311.94±13.84	311.20±11.56	-0.357	0.722 a	-0.058 (-0.378-0.262)
Outer retinal thickness (μm)	279.98±10.50	276.77±11.14	-1.818	0.071 a	-0.297 (-0.618-0.025)

GCL, Ganglion cell layer thickness; IPL, Inner plexiform layer thickness; Measurement data with a normal distribution are expressed as "mean ± standard deviation"; Measurement data with a non-normal distribution are expressed as "median (range)". a represents the independent samples t-test, and b represents the Mann-Whitney U test.

Compare the RNFL thickness and macular parameters between the ADHD group and the control group. The results showed that in both the ADHD group and the control group, the retinal thickness conforms to the normal valley-like macular structure with a thinner centre and thicker periphery. There were no statistically significant differences in the average macular area thickness, central macular retinal thickness, inner and outer ring retinal thickness, and the thickness of the inner plexiform layer of ganglion cells in the macular area between the ADHD group and the control group (all *p* > 0.05) (**Table 2**).

### 3.3 Comparison of VEP waveforms between the two groups

**Figure 1:**
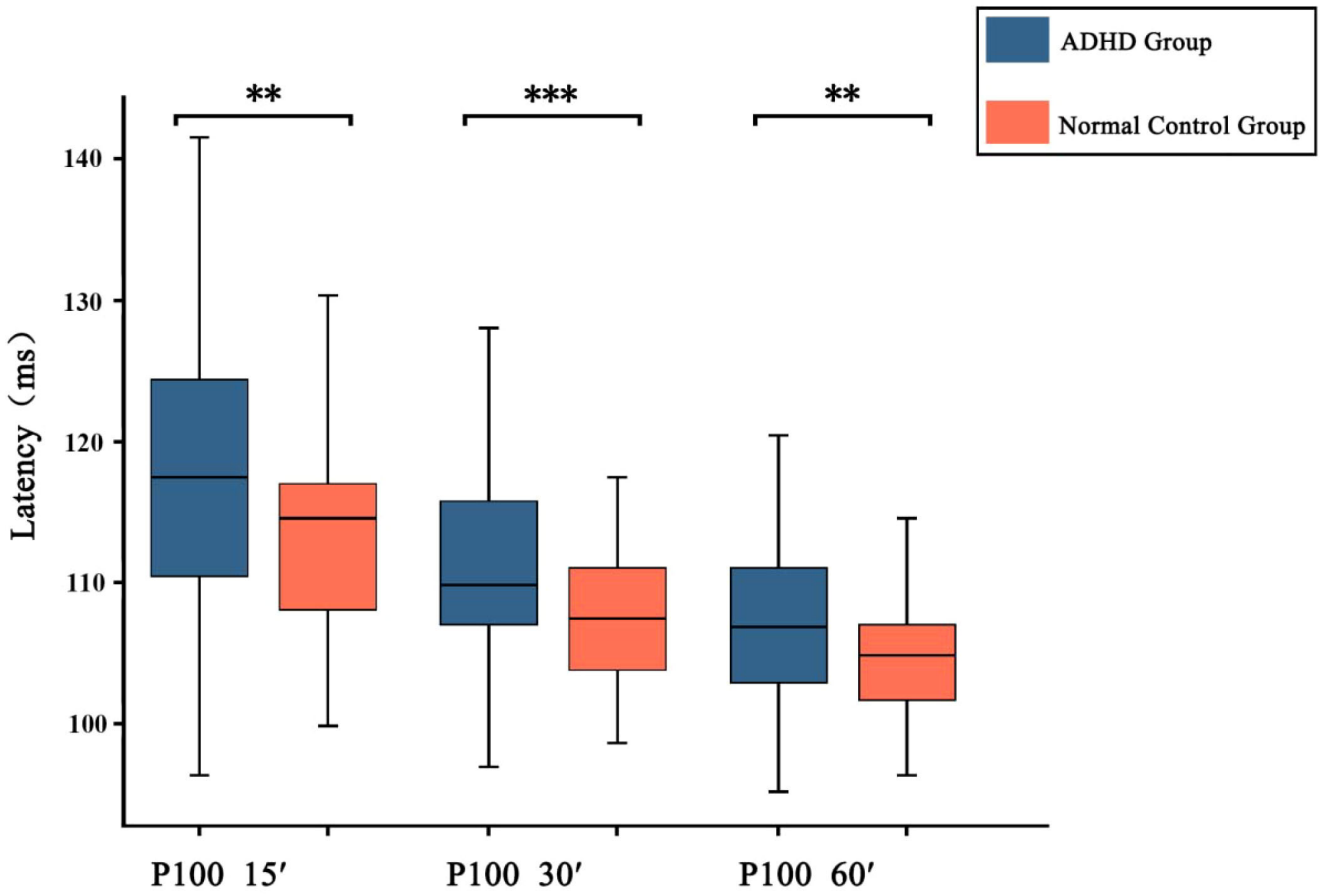
The latency of the P100 wave in two groups of children changes with the spatial frequency. In the statistical results, *p* < 0.001 is marked as ***, and 0.001 < *p* < 0.01 is marked as **.

The Mann-Whitney U test was used to compare the P100 latency and amplitude of the ADHD group and the control group at three different spatial frequencies (visual angles of 15′, 30′, and 60′). The results showed that the P100 wave could be induced in both groups under the three different spatial frequency stimuli. And as the spatial frequency increased, the latency gradually prolonged. Comparing the ADHD group with the control group, the results showed that the P100 latency in the ADHD group at the three spatial frequencies was statistically significantly longer than that in the control group (P100 latency for 60′: Z = -2.611, *p* = 0.009; P100 latency for 30′: Z = -3.546, *p* < 0.001; P100 latency for 15′: Z = -2.781, *p* = 0.005) (**Figure 1**).

**Figure 2:**
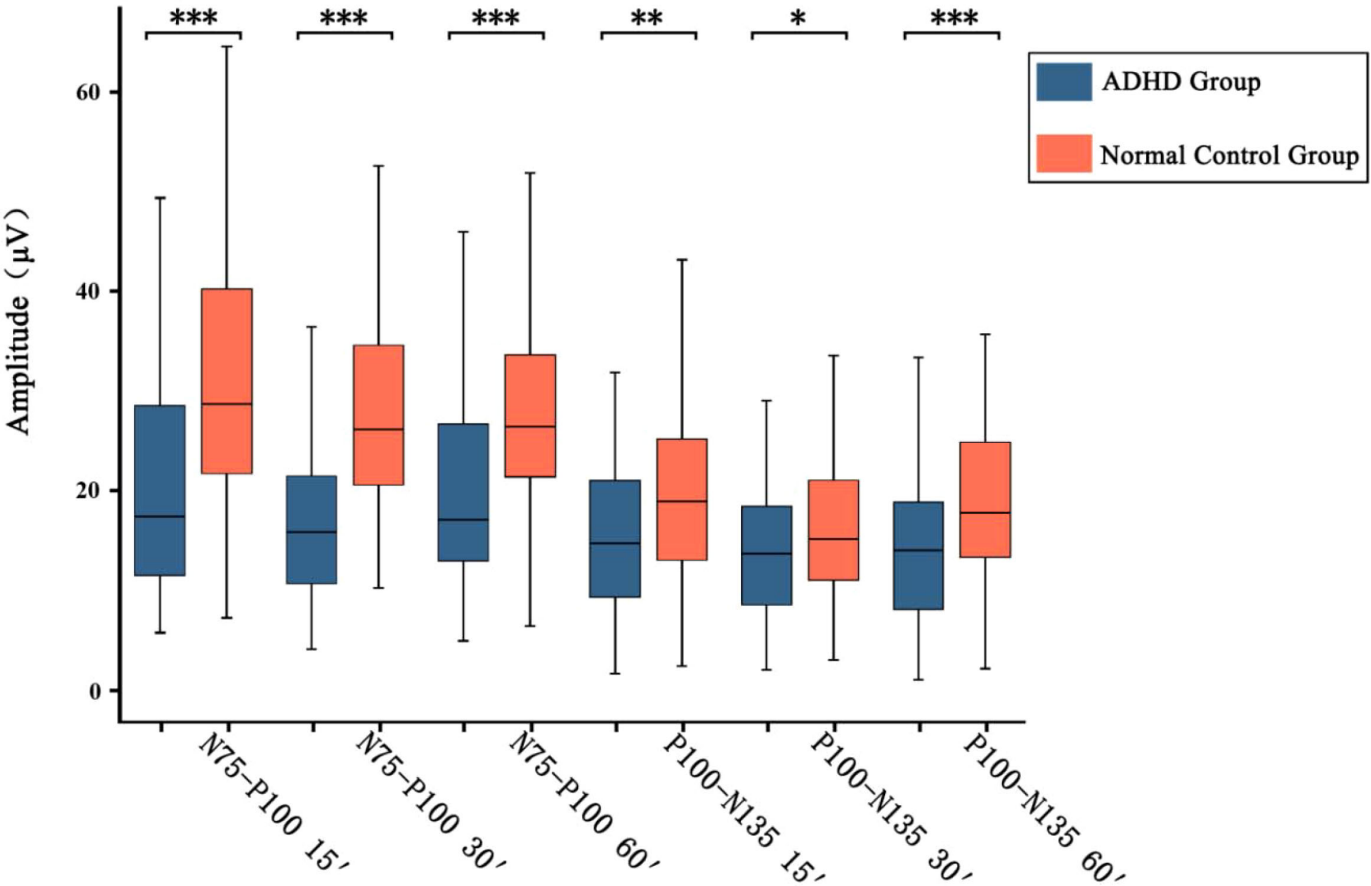
The changes in the amplitudes of the P100 waves in two groups of children with spatial frequency. In the statistical results, *p* < 0.001 is marked as ***, 0.001 < *p* < 0.01 is marked as **, and 0.01< *p* <0.05 is marked as.

The results showed that as the spatial frequency increased, both the amplitudes of N75-P100 and P100-N135 waves gradually decreased. Comparison between the ADHD group and the control group revealed that the amplitudes of N75-P100 and P100-N135 waves at three spatial frequencies in the ADHD group were statistically significantly lower than those in the control group (Amplitude of N75-P100 at 60′: Z = -4.947, *p* < 0.001; Amplitude of P100-N135 at 60′: Z = -3.481, *p* < 0.001; Amplitude of N75-P100 at 30′: Z = -5.812, *p* < 0.001; Amplitude of P100-N135 at 30′: Z = -2.051, *p* = 0.040; Amplitude of N75-P100 at 15′: Z = -4.733, *p* < 0.001; Amplitude of P100-N135 at 15′: Z = -3.210, *p* = 0.001) (**Figure 2**).

### 3.4 Correlation between VEP waveforms and SNAP-IV rating scale scores in the ADHD group

**Figure 3:**
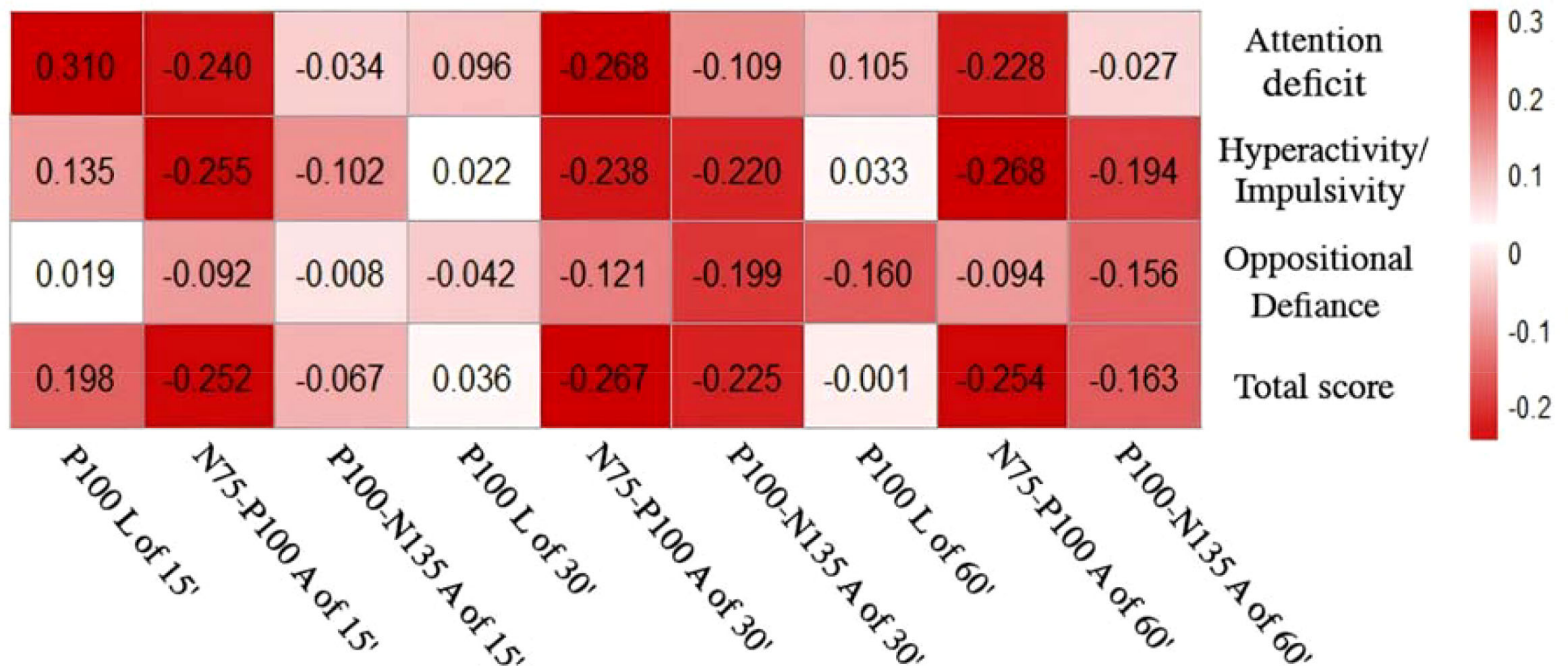
Correlation between VEP waveforms and SNAP-IV rating scale scores in the ADHD group.

Spearman correlation analysis was performed between the latency of P100 wave, the amplitude of N75-P100 wave, and the amplitude of P100-N135 wave in the ADHD group and the scores of the SNAP-IV rating scale (scores of the three sub-scales of inattention, hyperactivity/impulsivity, and oppositional defiance, and the total scale score). The results showed that: the inattention sub-scale score in the ADHD group was correlated with the latency of P100 wave at 15′ (*r* = 0.310, *p* = 0.007), the amplitude of N75-P100 wave at 15′ (*r* = -0.240, *p* = 0.04), and the amplitude of N75-P100 wave at 30′ (*r* = -0.268, *p* = 0.021); The hyperactivity/impulsivity sub-scale score was correlated with the amplitude of N75-P100 wave at 15′ (*r* = -0.255, *p* = 0.028), the amplitude of N75 -P100 wave at 30′ (*r* = -0.238, *p* = 0.041), and the amplitude of N75-P100 wave at 60′ (*r* = -0.268, *p* = 0.021); The total score of the SNAP-IV rating scale was correlated with the amplitude of N75-P100 wave at 15′ (*r* = -0.252, *p* = 0.030), the amplitude of N75-P100 wave at 30′ (*r* = -0.267, *p* = 0.021), and the amplitude of N75-P100 wave at 60′ (*r* = -0.254, *p* = 0.029) (**Figure 3**).

## 4 Discussion

This study demonstrated the structural and functional conditions of the retina and visual pathway in children with ADHD through OCT and VEP, and compared them with those of healthy controls. Overall, although there was no significant change in the RNFL in children with ADHD, there might be suggestive of reduced rim area. In children with ADHD, the latency of the P100 wave was significantly delayed and the amplitude was significantly reduced at different spatial frequencies.

This study compared various parameters between ADHD group and healthy controls. We found that there were no significant differences in the average macular thickness, central macular retinal thickness, inner ring and outer ring retinal thickness, and the thickness of the inner plexiform layer of ganglion cells in the macular area between these two groups. The results of this study are consistent with the macular structure of normal individuals.

In recent years, only limited studies have explored the RNFL thickness in ADHD group. Li et al. (18) conducted a systematic review and meta-analysis in children to investigate the relationship between RNFL thickness and ADHD. A total of four studies were included in this research, which showed that although there was a reduction in the overall RNFL thickness of ADHD group, the reduction was slight compared with that of control children. Due to the limited research in this field, the sample size included in this study was relatively small. While all four included studies were published in Turkey, racial differences may need to be considered as an influencing factor. Although all four studies used the spectral-domain OCT (SD-OCT) method, different instruments were used: two studies used the Heidelberg Spectralis, one study used the Cirrus OCT, and one study used the Opto Vue. These differences may all lead to biases in the research results. Contrastly, the study by Sima et al. (12) showed that there were no significant differences in the RNFL, ganglion cell complex, and macular thickness between ADHD group and healthy controls. In our study, the overall RNFL thickness of ADHD group was 102.7 ± 10.1 um, while that of healthy controls was 103.7 ± 7.0 um, which was very similar to the average RNFL thickness (95.6 ± 8.7 um) reported by Al-Haddad et al. (14). Our results were different from those of Li et al. (18), but consistent with the review by Sima et al. (12), indicating that there was no statistically significant difference in the overall RNFL thickness between ADHD group and healthy controls.

We propose that the following three reasons may account for the lack of significant difference in the RNFL thickness between ADHD group and healthy controls in this study. Firstly, although the exact aetiology of ADHD remains unclear, it most likely has a multifactorial origin. Previous studies have shown that thinning of the macula and RNFL in patients with neurodegenerative diseases, such as Parkinson’s disease, Alzheimer’s disease, and multiple sclerosis, is considered to be the result of the loss of neurons and axons of ganglion cells (41). The reduction in RNFL thickness may reflect neuronal atrophy in neurodegenerative diseases and may be related to neurodegenerative changes. Apoptosis occurs in the retinal ganglion cells of neurodegenerative diseases, which may lead to anterograde deterioration of the visual pathway, resulting in a thinner RNFL and ultimately leading to atrophy of the occipital visual cortex. Conversely, damage to the visual cortex may lead to retrograde deterioration of the visual pathway, ultimately resulting in changes in the optic nerve and retinal layers (12). For example, thinning of the RNFL and ganglion cell layer and changes in retinal volume in Alzheimer’s disease (15), multiple sclerosis (10), and Parkinson’s disease (11). Retinal parameters are used as structural indicators for axonal malformation and neurodegenerative diseases. Our study found that there was no significant difference in the thickness of the inner plexiform layer of ganglion cells in the macular area between ADHD group and healthy controls (15). One possible explanation is that ADHD is not a neurodegenerative disease, and the retinal characteristics of neurodevelopmental disorders may be different (12). Secondly, although SD-OCT can capture images at high speed, ADHD group have poor compliance compared with healthy controls. As a result, children with moderate to severe ADHD may be excluded from the study due to poor cooperation (18). According to the scoring results of the SNAP-IV scale in our study, the scores of the two sub-scales of attention deficit and hyperactivity/impulsivity symptoms and the total scale score of ADHD group were statistically significantly higher than those of healthy controls. However, the degree of attention deficit was mildly abnormal, that is, these were ADHD group with mild symptoms (16). Therefore, the overall reduction degree of RNFL in ADHD group may be underestimated. Thirdly, in some neuropsychiatric diseases, such as schizophrenia (42) and bipolar disorder (43), an inverse relationship has been found between the duration of the disease and the thickness of the RNFL as well as the macular thickness. Neurotransmitters such as dopamine, glutamate, and gamma-aminobutyric acid are essential for the development of post-ocular structures such as the thalamus and visual cortex. In the prefrontal cortex and striatum of patients with ADHD, there are decreased levels of gamma-aminobutyric acid, increased levels of glutamate, and dysregulation of neurotransmission in the dopaminergic pathway. It is reported that dopamine has multiple trophic effects on retinal function. Some studies suggest that the decrease in dopamine content in patients may leads to the loss of retinal ganglion cells, resulting in a thinner RNFL. As a neurotoxin, glutamate may also cause the destruction and loss of retinal ganglion cells and lead to the atrophy of the inner retina (38). We propose that due to the chronic and persistent neurotransmitter dysfunction, adult patients with ADHD are more likely to experience changes in RNFL thickness than ADHD children. The study by Münevver et al. (43) also certainly supported our suggestion. Their study results indicate that in adult ADHD cases, the RNFL thickness is thinner than that in normal individuals. According to approximately 50% of ADHD children will have persistent symptoms into adulthood, and the symptoms of ADHD present a persistent and chronic course (44, 45). Therefore, considering the onset time and symptom duration of adult ADHD, it may take several years for RNFL thinning to occur in ADHD. There may be some differences in RNFL thickness between ADHD adults with long-term persistent symptoms and ADHD children. This explanation should be considered preliminary and requires more future research for confirmation.

The results of this study found that the optic disc area, average cup-to-disc ratio, and optic cup volume of ADHD group were all statistically larger than those of the control group. Although there was no significant difference in the rim area between ADHD group and healthy controls, the rim area of ADHD group showed a suggestive shrink. Previous studies have suggested that before the thinning of the RNFL thickness, there is first a reduction in the rim area, followed by an increase in the cup volume and cup-disc ratio. This may be due to the loss of axons of optic nerve ganglion cells, which leads to a decrease in the amount of nerve tissue in the optic disc rim (6, 10, 11). Therefore, the results of this study suggest: although there are no statistically differences in various parameters between ADHD group and healthy controls, such as the average RNFL thickness, the nerve fibre layer thickness in the superior, inferior, temporal, and nasal quadrants of the optic disc, and the retinal thickness in the macular area. However, children in the early stage of ADHD may already show a suggestive reduction of optic disc rim area, and varying degrees of enlargement in optic disc area, cup-to-disc ratio, and optic cup volume. In the next step of the research, we need to include a larger sample size and a longer follow-up time to obtain more accurate results.

Subsequently, we also compared the latency and amplitude of the P100 wave between two groups at three different spatial frequencies (visual angles of 15′, 30′, and 60′). The results showed that the P100 wave could be induced in all participants at three different spatial frequencies. Compared with healthy controls, the latency of the P100 wave induced in ADHD group at three different spatial frequencies was significantly prolonged. The amplitudes of the N75-P100 wave and P100-N135 wave induced in ADHD group at three spatial frequencies were significantly lower than those in healthy controls. Previous studies have found that different neuroelectrophysiological signals can be detected by VEP in children with different neurodevelopmental disorders (32, 46). Zhang Kaifeng et al. (33) detected VEP waveforms in children with learning difficulties and healthy controls at five different spatial frequencies (visual angles of 108′, 54′, 27′, 13′, and 7′). The results showed that P100 waves could be evoked in both groups at five spatial frequencies. Moreover, as the spatial frequency increased, the latency gradually prolonged approximately linearly, and the amplitude of P100 also gradually decreased. Compared with healthy controls, the latency of P100 in children with learning difficulties was significantly prolonged at five spatial frequencies, and the prolongation was more significant at medium and high spatial frequencies; the amplitude of the P100 wave was also significantly lower than that of healthy controls. Our results show close agreement with those of Zhang Kaifeng et al. (33), which suggested that the visual information processing ability of ADHD group may vary at different spatial frequencies.

Only limited studies have attempted to conduct electrophysiological research on patients with ADHD through the characteristics of VEP signals. Our research results are consistent with those of Alaa et al. (34). Their results showed that both children with ASD and those with ADHD had a more obvious delay in the latency of the P100 wave compared with the control children. The results show that children with neurodevelopmental disorders may have delayed nerve conduction velocity, which is manifested as prolonged latency of the P100 wave in the VEP waveform. The VEP is almost the only neuroelectrophysiological tool for studying the early responses of the visual conduction pathway. The latency of the P100 wave, which is the time required for visual impulses to conduct in the visual pathway, can indirectly reflect the degree of lesions in the myelin and axons of the optic nerve and the speed of nerve conduction. The amplitude of the P100 wave reflects the number of nerve fibres involved in visual excitation and can indirectly reflect the degree of damage to the optic nerve axons and the structure and function of ganglion cells (20).

In this study, the latency of P100 in ADHD group was more significantly prolonged at medium and high spatial frequency visual stimuli. Since the higher the spatial frequency of visual stimuli, the smaller the visual angle, the lower the spatial resolution of the picture, and the more complex the visual information. It suggests that ADHD group have more difficulty in processing complex information and slower nerve fibre conduction velocity. Moreover, the amplitudes of N75-P100 and P100-N135 evoked by three spatial frequency stimuli in children with ADHD were significantly lower than those in normal control children. It is possible that in ADHD children, when processing visual information, the number of retinal ganglion cells involved in visual information processing is relatively small, and the number of optic nerve axons or the neurons generating evoked potentials is reduced. As a result, the conduction speed of visual information in children with ADHD slows down, the reaction time for nerve impulses to reach the visual cortex is prolonged, and the amplitude of the P100 wave decreases. We suggest that due to the dysfunction of the dopaminergic pathway in patients with ADHD and the degeneration of dopaminergic neurons in the retina, the production and secretion of dopamine are reduced (47). It is plausible that this may affect the functions of cells in the inner plexiform layer and horizontal cells in the retina, thereby disrupting the transmission of visual signals and leading to the delay of the latency and the reduction of the amplitude of VEP P100 (48). In addition, we also investigated whether there was a correlation between the latency of the P100 wave, the amplitude of the N75-P100 wave, and the amplitude of the P100-N135 wave in ADHD group and the scores of the SNAP-IV rating scale. The results showed that: there was a negative correlation between the total score of the SNAP-IV rating scale and both the amplitude of the N75-P100 wave and the amplitude of the P100-N135 wave. And there was a positive correlation between the total score of the SNAP-IV rating scale and the latency of the P100 wave. That is, the more severe the ADHD symptoms, the lower the amplitude of the P100 wave and the longer the latency of the P100 wave. This indicates that although currently the results of VEP cannot be used as a specific biological marker for the diagnosis of ADHD, they have a certain evaluation value for the severity of the disease.

## 5 Limitations of the study

Some aspects of our study require some caution when interpreting the results. Firstly, our research is an exploratory study with a relatively limited sample size, including 37 children with ADHD and 38 healthy controls. Including a larger sample size and a longer follow-up period may lead to more accurate results. Secondly, although the VEP and OCT methods we used are rapid and non-invasive detection techniques, they still require a certain degree of cooperation from the participants. This may lead to the exclusion of children with moderate to severe ADHD due to their poor compliance. The heterogeneity of children with ADHD may be underestimated. Thirdly, if the changes caused by ADHD require long-term effects, this may mean that such changes are more obvious in adult ADHD patients. The participants in our study were children with ADHD, which may lead to an underestimation of the heterogeneity of ADHD patients. Fourthly, our research is an exploratory study, and previous studies are only similar but not exactly the same. As a result, we did not conduct *a priori* power analysis before the study.

## 6 Conclusions

In general, there is mostly non-significant structural alteration in ADHD children. However, children with ADHD may already show a suggestive reduction of optic disc rim area, and varying degrees of enlargement in optic disc area, cup-to-disc ratio, and optic cup volume. More importantly, in children with ADHD, the latency of the P100 wave at three different spatial frequencies was significantly prolonged, and the amplitudes of the N75-P100 wave and the P100-N135 wave were significantly reduced. In conclusion, in the study of the structural and functional changes of the optic nerve pathway in ADHD group, the examination of the latency and amplitude of the VEP P100 wave may be more sensitive than the morphological examination such as the RNFL thickness. Although currently the results of VEP cannot be used as a specific biological marker for the diagnosis of ADHD, they have a certain evaluation value for the severity of the disease. From the perspective of electroencephalophysiology, this study further suggested that ADHD children may have early visual information processing disorders, providing certain theoretical and practical value for further early intervention in ADHD children from the field of visual perception.

## Data Availability

The original contributions presented in the study are included in the article/supplementary material. Further inquiries can be directed to the corresponding authors.
